# Enhancing community weight loss groups in a low socioeconomic status area: Application of the COM‐B model and Behaviour Change Wheel

**DOI:** 10.1111/hex.13325

**Published:** 2021-08-04

**Authors:** Nia Coupe, Sarah Cotterill, Sarah Peters

**Affiliations:** ^1^ Faculty of Health and Medicine, Lancaster Medical School Lancaster University Lancaster UK; ^2^ Faculty of Social Science, School of Psychology University of Chester Chester UK; ^3^ Division of Population Health, Health Services Research and Primary Care, Faculty of Biology, Medicine and Health, School of Health Sciences, Centre for Biostatistics The University of Manchester Manchester UK; ^4^ Division of Psychology and Mental Health, Faculty of Biology, Medicine and Health, School of Health Sciences, Manchester Centre for Health Psychology The University of Manchester Manchester UK

**Keywords:** COM‐B, goal setting, intervention design, low socioeconomic status, obesity, stakeholder

## Abstract

**Background:**

Obesity rates are higher among people of lower socioeconomic status. While numerous health behaviour interventions targeting obesity exist, they are more successful at engaging higher socioeconomic status populations, leaving those in less affluent circumstances with poorer outcomes. This highlights a need for more tailored interventions. The aim of this study was to enhance an existing weight loss course for adults living in low socioeconomic communities.

**Methods:**

The Behaviour Change Wheel approach was followed to design an add‐on intervention to an existing local authority‐run weight loss group, informed by mixed‐methods research and stakeholder engagement.

**Results:**

The COM‐B analysis of qualitative data revealed that changes were required to psychological capability, physical and social opportunity and reflective motivation to enable dietary goal‐setting behaviours. The resulting SMART‐C booklet included 6 weeks of dietary goal setting, with weekly behavioural contract and review.

**Conclusion:**

This paper details the development of the theory‐ and evidence‐informed SMART‐C intervention. This is the first report of the Behaviour Change Wheel being used to design an add‐on tool to enhance existing weight loss services. The process benefitted from a further checking stage with stakeholders.

## 1 INTRODUCTION

The increased health risks, lower health quality of life and psychosocial consequences associated with obesity^1^ mean that it is one of the world's major public health challenges. Global rates of obesity and overweight are rising^2^ particularly among people of lower socioeconomic status (SES) living in middle‐ to high‐income countries,^3^, ^4^ and in certain deprived UK communities.^5^ A recent report described the evidence supporting an inverse relationship between SES and obesity as ‘overwhelming’^6^ (p. 7); socioeconomic indicators such as occupational social class, education attainment, income and area‐level deprivation have been strongly associated with the risk of obesity for adult populations in the United Kingdom.^7^, ^8^ Low SES individuals are less likely to engage in the behaviours associated with maintaining a healthy weight.^9^, ^10^, ^11^, ^12^ Furthermore, weight loss intervention trials are less likely to recruit, retain and engage lower SES groups.^13^, ^14^, ^15^, ^16^, ^17^ This body of evidence suggests that a ‘one size fits all’ approach is not only ineffective for these populations, but may even increase health inequalities. Indeed, social inequality in the prevalence of obesity is predicted to widen,^18^ and the increased severity and poorer outcomes associated with obesity and coronavirus disease 2019 may contribute further.^19^ While there is no single solution to addressing this, one element of a whole systems approach is to ensure that health behaviour interventions delivered to this population are appropriately tailored to their specific needs.^20^

Previous work has identified barriers around delivering a generic intervention to diverse groups, given varying levels of knowledge, language and literacy skills and cultural considerations.^21^ Other barriers to successful interventions in this population include access to healthy and unhealthy foods, and leisure facilities,^21^, ^22^ neighbourhood safety concerns,^23^ complex social situations and social norms.^21^ Actual and perceived cost of healthy foods and physical activity is also an important consideration for this population,^21^, ^24^ and addressing price concerns in tailored interventions may be important.^25^

The term ‘stakeholder engagement’ refers to the involvement of all decision makers in research, including health professionals and public, and their involvement as contributors has the potential to increase research quality and impact.^26^ Indeed, involving stakeholders in intervention design is recommended as it not only increases understanding of context but also increases the chance of success at later evaluation and implementation stages.^27^, ^28^ As well as involving stakeholders, the application of theory in intervention design is important to ensure that key determinants are targeted.^27^ The Behaviour Change Wheel (BCW)^28^ was designed to merge common components of behaviour change theories and link them with a broad model of behaviour: the COM‐B. This model is integral to the BCW; it identifies the areas that result in behaviour change and that should be the focus of intervention: Capability (Psychological & Physical), Opportunity (Physical & Social) and Motivation (Reflective and Automatic). The BCW has informed interventions in various health contexts, and reported to be effective in changing the target behaviours.^29^, ^30^ As yet, the BCW has not been applied to the context of low SES populations. The aim of this study was to use the BCW^28^ alongside stakeholder involvement (staff and public) to design an intervention (SMART‐C). The intervention aims to enhance adherence and engagement outcomes to promote weight loss in low SES populations.

## 2 METHODS

**Figure 1:**
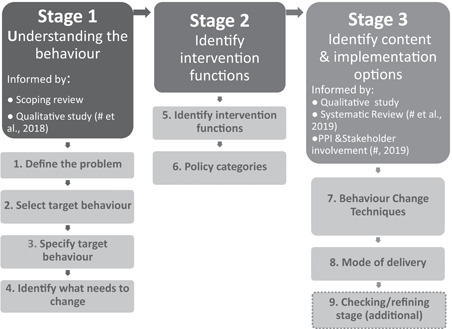
Stages of the Behaviour Change Wheel in the design of the SMART‐C intervention

The intervention design process was guided by the BCW,^28^ along with Medical Research Council guidance on complex intervention design.^31^ The BCW is comprised of three stages, each of which was informed by research (Figure 1).

The research team met every 2–4 weeks over 2 years (2015–2017) to discuss the development of the intervention and associated work. The team consisted of a doctoral‐level student (lead author) supervised by two senior academics based within the same institution (co‐authors). The lead author attended a week‐long training on the BCW provided by its authors, further training on behaviour change techniques (BCTs) and had a range of experience in qualitative methods and research in health services research. The supervisors had experience and expertise in health psychology, health services research, behaviour change techniques and qualitative and quantitative methods for developing and testing complex interventions.

### 2.1 Stage 1: Understanding the behaviour

#### 2.1.1 Define the problem in behavioural terms

To identify the problem in terms of the behaviour that the intervention will be targeting, a combination of a scoping review and qualitative interviews and observations^31^ within the selected setting was used.

##### (a) Literature review

A scoping review was undertaken to identify key determinants in relation to weight‐related behaviour change within low SES communities, identify research gaps and to inform our qualitative study. Relevant databases were searched (Medline, Embase, CINHAL, PsycInfo, Web of Knowledge) to identify qualitative and quantitative research using a combination of key terms relating to low SES populations, obesity/overweight, behaviour change and weight loss.

##### (b) Setting and qualitative study

Research in weight loss groups in a low SES city in the North West of England was undertaken, chosen based on its level of deprivation; it is among the 20 local authorities with the highest proportion of neighbourhoods in the most deprived 10% of neighbourhoods in England.^32^ The weight loss groups were run by the local authority, delivered weekly in community venues over a minimum of 6 weeks, with an average 40%–55% course completion rate before 2020. The groups were aimed at anyone seeking support around weight control, were free to attend and accessed through self‐referral or GP‐referral. Group content was developed and delivered by experienced, non‐clinical staff, covering the following evidence‐based topics: the Stages of Change Model,^33^ the Eatwell plate,^34^ understanding food labels, SMART goals and portion sizes, meal planning, overview of fats and sugars, benefits of exercise and alcohol consumption. Attendees were weighed each week and provided information around these topics, which were discussed through group activities.

Qualitative data collection comprised observations of four weight‐loss groups, and semi‐structured interviews with 11 service facilitators (10 females), all involved in delivering health promotion advice across the city, and 14 service users (13 female). The average age of the service users was 66 years (44–84), and the majority (13) were White British. All facilitators delivering the groups were interviewed, and the service user sample was broadly representative of those attending the groups, except for two groups that targeted and were tailored to specific cultural communities (Yemeni and Jewish women). Interviews explored views on the local area, current provisions within the community/group, area‐specific factors contributing to obesity and weight loss experiences. Further details, including the full inductive thematic analysis^35^ results, have been reported elsewhere.^21^

#### 2.1.2 Select and specify target behaviour

While Stage 1 helps understand the context and overall issue to be tackled within an intervention, this second stage ensures that these behaviours are explored in more detail to identify very specific behaviours that needed to be targeted. We then specified who needed to perform these behaviours (the service users and/or the facilitators); what they needed to do; and when, where, how often and with whom it should be performed.

#### 2.1.3 Identify what needs to change

This step comprised two distinct phases to identify what exactly needs to change in relation to the COM‐B model for the behaviour to occur. First, a deductive content analysis^36^ was conducted, whereby themes from the inductive thematic analysis^21^ relating to the target behaviour were mapped onto the COM‐B constructs: psychological/physical capability, social/physical opportunity and reflective/automatic motivation.^28^ A second expert rater coded 20 excerpts from the transcripts, with substantial agreement (Cohens kappa 0.78). Data were revisited to check for any other themes relating to these constructs that may not have been identified in the inductive analysis. We then performed the behavioural diagnosis as per the BCW guidance,^37^ which involves identifying what needs to happen for the target behaviour to occur within each component of the COM‐B and whether or not there is a need for change to the current situation (e.g., more knowledge required suggests a need to target psychological capability in the resulting intervention). The behaviours were mapped onto both the sources of behaviour (Capability, Opportunity, Motivation) and the theoretical domains framework (TDF) domains,^38^, ^39^ as the TDF can provide a more detailed understanding.

### 2.2 Stage 2: Identify intervention functions

#### 2.2.1 Identify intervention functions

The BCW guide includes nine intervention functions (education, persuasion, incentivization, coercion, training, restriction, environmental restructuring, modelling and enablement) linked with the COM‐B and TDF to facilitate the identification of the most suitable and likely effective intervention function(s) in any given context. The intervention functions were selected based upon the outcome of the behavioural diagnosis, whereby the COM‐B component was identified and the guidance was used to identify the relevant intervention functions. The APEASE criteria evaluation (affordability, practicability, effectiveness, acceptability, side‐effects and safety and equity) was applied to identify which would be most viable.^37^ This was completed initially by the lead author, and agreed by regular discussions with the co‐authors.

#### 2.2.2 Policy categories

This step identifies the most appropriate policy category from the final wheel of the BCW (fiscal measures, guidelines, regulation, environmental/social planning, communication/marketing, service provision, legislation).

### 2.3 Stage 3: Identify content and implementation options

#### 2.3.1 Behaviour change techniques

Behaviour change interventions are constructed of bundles of BCTs, which are the smallest components of an intervention, for example, goal setting or action planning. While the BCW provides guidance on the most commonly used BCTs, it does not provide information on efficacy.^37^ Given the focus of the scoping review and qualitative study on adherence and lack of commitment to behaviour change in groups, we conducted a systematic review focused on ‘commitment devices’ to determine efficacy.^40^ Broadly speaking, this refers to BCTs concerned with increasing an individual's commitment to a particular goal such as commitments, pledges and behavioural contracts.

#### 2.3.2 Mode of delivery

This stage involves identifying the best mode of delivery of the intervention, such as phone apps, internet and posters, among others, and relies on whether intervention delivery will be face to face or distance, and at an individual, group or population level. The APEASE criteria were also used to select the most suitable mode.^37^

#### 2.3.3 Checking stage

Service facilitators and users checked usability and content appropriateness of the first iteration of the new intervention. The SMART‐C intervention was sent to all facilitators involved in the initial qualitative study a week in advance of a face‐to‐face meeting. Of the 11 facilitators invited, three (all female) attended the meeting, where feedback was sought through group discussion. Those who could not attend were invited to provide feedback by email, though none was received. Ten service users (seven female) were given SMART‐C at a weight loss group during a week where the usual facilitator was unable to attend. They were given time to work through the booklet, followed by individual and group discussions with the researcher. All stakeholders were encouraged to provide feedback (both positive and negative) and to make suggestions for changes. Further details about this study and its impact are reported elsewhere.^41^

## 3 RESULTS

### 3.1 Define the problem in behavioural terms

The initial problem as outlined in the introduction was that weight loss interventions tend not to effectively engage people in low SES areas compared to more affluent populations, whose adherence is reported to be higher.^14^, ^15^, ^16^, ^17^ Our previous work suggested that the focus should be on changing dietary behaviours, given that many had already incorporated physical activity into their lives and were well supported in this service.^21^ For example, one service user identified that ‘there's plenty to do that caters for everybody’ (SU1) such as walking groups, cycling groups and over 50 s Zumba, either free or provided at a reduced rate. Service users were given a pass to attend the gym or swimming pool for free during the course. This was described as ‘like a carrot you can dangle for them’ (F1) to encourage physical activity, and was perceived as successful:

it's quite a good way of getting people, encouraging people to exercise and increase physical activity and then from there you know some of them decide to get a membership there as well. (F2)

However, making changes to dietary habits proved more problematic, with facilitators identifying healthy eating as the ‘one to focus on’ (F2) in relation to the intervention. Therefore, the overall problem behaviour that we were addressing was poor adherence to dietary elements of lifestyle interventions by adults with obesity/overweight within low SES areas.

### 3.2 Select and specify the target behaviour

The qualitative data revealed two levels of behaviour to target: Dietary behaviours and in‐group behaviours. Across the data sources (qualitative interviews, observations and scoping review), four dietary behaviours were identified: (A) decrease portion sizes, (B) use food labels to improve food purchasing, (C) increase fruit and vegetable consumption and (D) decrease energy‐dense fast food consumption. The in‐group behaviour identified was that service users (SUs) did not engage with goal‐setting activities, setting vague or unrealistic goals, or no goals at all during the groups, despite goal setting being part of the course:

I don't think [the facilitator] did [set any goals]…I don't think she did a goal weight. I don't know. (SU9)

With its roots in organisational psychology,^42^ goal setting has been identified as effective across a broad range of behaviours and identified as a potential essential component of behaviour change interventions.^43^ Importantly, goal setting has been identified as effective in changing dietary behaviours, specifically in promoting healthy eating amongst adult populations with overweight and obesity.^44^ As such, we decided to focus on improving SUs' engagement with setting SMART (Specific, Measurable, Achievable, Realistic, Timely) goals. Furthermore, facilitators did not actively check the suitability of the goals: ‘I don't check it for them unless they want to bring it forward’ (F1). Given that unsuitable goals were often being set, both unspecific and over ambitious, we decided to target facilitator behaviour to support SUs' goal setting. In addition, goals were rarely reviewed or revisited by facilitators, which we identified as a barrier to changing dietary behaviours, given that SUs reported having ‘no incentive to do it [healthy eating] properly’ (SU8). Therefore, we specified one target behaviour for the service users (choose and record a SMART dietary goal each week) and two for the facilitators (check goal suitability and goal adherence each week).

### 3.3 Identify what needs to change: COM‐B analysis

The objective of this analysis was to understand the barriers faced by SUs and facilitators in carrying out the goal‐setting behaviours, which were not routinely completed despite being part of the original course. As we were also interested in ensuring that goals were achievable, we included data relating to goal implementation in the analysis for example, barriers to enacting dietary goals. These barriers are described below in relation to the COM‐B constructs of Capability, Opportunity and Motivation and the TDF domains, and are summarised in Table 1.

**Table 1:** Intervention component matrix

Barriers to dietary goal setting & review in a low socioeconomic community weight loss group	COM‐B components	Theoretical domains framework	Intervention functions	Behaviour change techniques	Description of behaviour change techniques within the SMART‐C intervention
Service user behaviour‐goal setting					
Lack of knowledge Poor knowledge and understanding around healthy diet Portion size: Knowing what a portion size should be Food labels: Understanding them and how to read them Fruit and vegetables: What they are and what counts as a portion Take away/fast food: How unhealthy they can be and what better options there are	Psychological capability	Knowledge	(Education already part of course) Enablement	Informed content of Goal setting (behaviour)	Goals were predefined to cover these four areas, so goal setting did not rely on SU knowledge (education already covered within the intervention)
Poor understanding of process Did not understand how to set SMART goals, or unable to choose a goal		Knowledge skill Memory, attention and decision processes	Enablement	Instruction on how to perform the behaviour *(goal setting)* Conserve mental resources	Intervention includes a page outlining how to set a SMART goal Goals predefined to narrow down choices
Language and literacy Could not write, or did not want to		–	Cognitive skills	–	Not addressed directly, informed design of intervention. Reduced text within booklet, little writing required, colour‐co‐ordinated pages, predefined goals to minimise writing
Resources Nowhere to write goals down	Physical opportunity	Environment‐al context and resources	Environmental Restructuring	Adding objects to the environment	Intervention designed in the form of a booklet with clear pages for goal setting
No incentive Not checked by facilitators	Reflective motivation	Beliefs about consequences	Enablement	Behavioural contract	Linking in with low motivation below, behavioural contract added to goal setting to ensure that the facilitator ‘checked up’ on the service user's goals
Service user behaviour‐goal implementation					
Remembering goals Where service users did not write goals down, they often did not remember what they had planned to do (memory) Specific to portion control: Specific to using food labels: Forget glasses to read	Psychological capability	Memory, attention and decision processes	Enablement Environmental restructuring (if prompts and cues)	Prompts and cues	Providing a booklet with space to keep track of goals to serve as a reminder and a prompt
Prioritising goals Service users had many competing interests, meaning even when goals had been set, they were not prioritised No time to implement some changes, e.g., weighing food for portions, checking labels in the supermarket	Psychological capability (cognitive load) Physical Opportunity (time)	Memory, attention and decision processes Environmental context and resources	Enablement Environmental restructuring	Action planning Conserving mental resources Prompts and Cues	Heuristic, easy to implement goals, not time consuming, Focus on one goals only to make more achievable, Set specific time/day/meal
Cost of goal implementation: Service users felt that they could not afford healthy food, e.g., fresh fruit and vegetables	Physical opportunity	Environmental context and resources	Enablement	Informed content of goal setting (behaviour)	Service already covering this, but used to inform content, e.g., cost consideration of goals
Low motivation For those without motivating events or health issues and given that the course was free, they had little motivation to make dietary changes, rather utilising the group as a social event	Reflective motivation	Goals Intentions	Incentivization	Behavioural contract Review behaviour goal	Add social consequence of behavioural contract and goal review
Facilitator behaviour‐actively check & review SU goals					
No time Facilitators did not have time to check everyone's goals individually, contributing to unsuitable goals being set	Physical opportunity	Environmental context and resources	Environmental restructuring	Prompts and cues	Contributed towards content of goal setting—predefined to reduce the amount of time spent required to assist Both the required signature and the review page also acted as prompts to remind the facilitator to check
Focus on service engagement: Facilitators stated that they were a ‘target driven service’ and required ‘bums on seats', as such facilitators did not like to ‘check‐up’ on service users, as they thought it may result in disengagement	Social opportunity/reflective motivation	Social influences Professional/social role & identity	Modelling Enablement	Demonstration of the behaviour Instruction on how to perform the behaviour Behavioural contract Review behaviour goals Prompt and cues	Facilitators were provided with a brief instruction manual that was discussed together Researcher attended weight loss groups and modelled the behaviour at the first session The booklet contains a section for both the service user and facilitator to sign, providing the facilitator opportunity to check Review section also acted as a prompt and provided an opportunity for facilitators to ‘check up’ on service user's behaviour

#### 3.3.1 Psychological capability (service users)

##### Knowledge

One barrier faced by some SUs in both setting suitable goals and implementing them was a lack of knowledge regarding what constitutes a healthy diet, despite this being covered within the course. For example, we observed a service user asking if a packet of crisps contributed towards their *‘*five‐a‐day’. Knowledge around fruit and vegetables was particularly highlighted in the facilitator interviews (‘people can get quite confused about what counts as a portion or as one of your five a day’ (F2) and that for some attendees ‘it's like starting from scratch’ (F9) in terms of their dietary knowledge. Another main issue was knowledge around meal portion sizes: ‘a lot of it is the portion control, is that they just don't know how much’ (F11) and literacy ([Sus aren't] ‘able to read food labels clearly and understand food labels’ (F6) and as such ‘are not aware of sugar and fats intake into the food’ (F10). This was identified as a barrier to implementing goals.

##### Skills

Facilitators identified that SUs struggled to set achievable goals and struggled to understand how to set SMART goals. It was observed that despite SUs being given instructions on what a SMART goal was and should comprise, they still set vague rather than specific goals (e.g., ‘eating healthier’. This is further supported by SU13: ‘they [staff] just say “think of three goals”, and then I put that, “losing 1kilo”…’. This meant that it was unclear if they knew how to implement these goals.

##### Memory/attention and decision processes

Service users often did not write goals down and plans were forgotten. This was unsurprising, given the complex social situations that were common amongst the SUs:

They just forget. A lot of the people that we work with have got a lot of other things going on in their lives, so, eating fruit, for example, during the week, even at the time they feel really committed, as soon as they walk out that door…it's completely out of their mind again. (F6)

##### Cognitive skills

Observations revealed that some SUs could not write down their goals due to limited literacy skills and/or language barriers (e.g., English as a second language). Facilitators described the SUs as having ‘very varying skills’ (F4), with one describing the literacy in their particular area as ‘an average age of nine’ (F7). This poor literacy was a clear barrier to recording weekly goals and to implementing some goals such as using food labels to guide their food purchasing and portion choices.

#### 3.3.2 Physical opportunity (facilitators and service users)

##### Environmental context and resources

Lack of time was an issue within the group in relation to ensuring that goals had been set. Facilitators tried to help when SUs struggled to set their own goals, but, as noted in the observations, time limits meant that they were largely unable to do this. This meant either no goal or an unrealistic one being set.

Time, or perceived lack of time, was also a barrier to implementation of goals, particularly in relation to portion control and weighing ingredients: ‘it's just that effort… and that time that we don't seem to think we have.’ (F11). Time was also a barrier in relation to reading food labels when shopping to help make better decisions about food purchasing:

how many of us go in a shop and stand there reading labels? Your shopping would take you four hours rather than an hour. (F5)

Observations revealed that SUs were not provided with pens or somewhere to write their goals each week, which was a barrier to setting dietary goals.

Participants highlighted the financial cost of goal implementation. Specifically, both real and perceived costs of fresh fruit and vegetables were barriers to purchasing, and therefore to increasing their consumption.

I started a healthy eating on a budget course because that's all I kept on getting from the lads that were there, well we can't afford to eat healthy ‘we'll just go and get a takeaway from round the corner'… (F12)

#### 3.3.3 Social opportunity/reflective motivation

##### Social influences

Due to the lack of knowledge identified, facilitators tended to focus the course on ‘educating’ (F3, F11) and reported being comfortable with a ‘no pressure’ (F1) approach, given that people were attending voluntarily. They were cautious not to disengage the SUs who were described as ‘very, very difficult to engage’ (F1) and ‘very hard to keep them engaged’ (F12). Furthermore, they described their service as ‘target driven’ (F1) in terms of attendance numbers, and were mindful of ‘trying to get them [SUs] to come back’ (F4). Facilitators were reluctant to be ‘over‐zealous and frighten them off’ (F4) or push SUs in case they failed or became disheartened. Some SUs appreciated this approach:

The good thing is they don't pressure you into doing anything, they give you lots of information, how to eat healthy, they don't call it a diet or anything like that, what's good foods and what are not so good foods, loads of literature if you want. (SU12)

Although this approach facilitated engagement with the service itself, this is also a barrier to dietary behaviour change; there was no expectation of commitment in the group and no incentives to make the dietary changes as SUs were not being ‘checked up’ (F1, SU8) on, or required to have filled in any forms.

#### 3.3.4 Reflective motivation (service user)

##### Intentions/goals

Service users with a health condition described being motivated to make dietary changes. For example, SU5 was motivating them to join the group and lose weight after being told by the GP that they ‘could end up having to go on insulin’. This was also reflected on by F2:

[SUs] that are pre‐diabetic or have diabetes … and have been told by the doctor that they need to lose some weight, I find that those people are quite determined to make the changes…but the people who dip in and out and their weight goes up and down. trying to get them to make a change to their lifestyle can be quite slow.

In the absence of health motivators, there appeared to be a lack of incentive to make necessary changes to adhere to their goals, particularly in the absence of any social influence within the group. For example, F9 stated that ‘everybody knows that they should do it, but people aren't motivated to do it’. Supporting this, SU3 had not identified any motivating factors such as health condition:

I do know what's right and what's wrong, it's just doing it…I wasn't in the mood for dieting because I think you've got to be motivated. If you've not got your mind set on it, it doesn't matter; you won't do it at all. (SU3)

Furthermore, many commented on the absence of an associated cost to the course like some more commercial courses (F1, F3, F4, F6, F7, F8, F9, F10). Although being free was identified as ‘essential to this area’ (F3), ensuring access to all, some highlighted that this also meant that SUs had less commitment to the course and less motivation to adhere to their goals.

### 3.4 Identify what needs to change: Behavioural diagnosis

The barriers to goal setting lay in the Psychological Capability of the service user's knowledge, skills and memory in relation to setting (and implementing) goals. Barriers were identified in relation to Physical Opportunity, specifically, time for facilitators to check the suitability of the goals and resources for SUs to write their goals. Reflective Motivation and social opportunity also required attention in relation to facilitators ‘checking up’ on service user behaviour and increasing SUs' motivation to make the changes. A summary of the links between what needed to change, the COM‐B components and TDF domains is presented in Table 1.

### 3.5 Identify intervention functions

The COM‐B analysis, and following the guidance, resulted in five main intervention functions being identified: Incentivization, Training, Modelling, Enablement and Environmental Restructuring.

### 3.6 Policy categories

Policy categories that matched our intervention functions included Guidelines, Regulation, Legislation and Service Provision. Given that we were already working with a service provider and were identifying ways to enhance the service, service provision was the most suitable in this situation.

### 3.7 Behaviour change techniques

An overview of the selected BCTs, with links to the sources of behaviour, TDF domains and intervention functions, is shown in Table 1. The final intervention SMART‐C (Supporting Information Material 1) comprised 6 weeks' worth of ‘commitment sheets’, with a choice of 10 goals (two options for each dietary behaviour), and space for a personalised goal, should none of the prespecified goals be appropriate. This included the four main dietary behaviours identified in Step 2 (decrease portion sizes, use food labels to improve food purchasing, increase fruit and vegetable consumption, decrease fast food consumption), and a further behaviour of increasing water consumption was added following feedback from stakeholders in the checking stage.

Predefined goals were included to tackle many of the barriers identified in the COM‐B analysis, including reliance on service user knowledge, skills in setting SMART goals, decision processes, reliance on the ability to write and lack of time (Table 1). Each goal is outlined in Table 2, and further details and rationales for these are included in Table 1.

**Table 2:** Predefined goal content and rationale

Predefined goals		Rationale
Portion Control Facilitators identified ‘portion size’ (F2, F9, F10) or ‘portion control’ (F4, F5, F11) as a major issue for weight management	A1: Use of smaller crockery	Targeting tableware is simple to implement, has no or low cost associated and reduces time spent measuring ingredients. A Cochrane systematic review concluded that swapping crockery is effective at reducing the quantity of food^45^ and has the potential for addressing ‘portion distortion’^46^
	A2: Use own hand as a reference for portion size	Based on an activity that already took place within the groups around using familiar objects as a short cut to measuring the portion sizes of different food groups. Based on similar guidance provided online by the British Heart Foundation and British Nutrition Foundation^47^, ^48^
Check food labels. Need to simplify to address literacy/language barriers	B1: Use food labels to improve food purchasing	This goal simplified the traffic light system, with the aim of choosing more green labels and reducing the number of red labels, as per the current UK government recommendations^34^
	Goal B2: Using food labels to reduce fat intake	The second goal focused specifically on fat content, aiming for less than 5%, which was already encouraged within the weight loss groups, and aligns with government recommendations (green = 3%, amber = 3%–17.5%).^49^ Focusing on only one part of the label had the potential to reduce the amount of time spent reading labels, which was identified as a barrier
Eat more fruit and vegetables Address limited knowledge around fruit and vegetables by providing prespecified goals	Goal C1: Add fruit and/or vegetables to diet	Limited evidence exists around increasing fruit and vegetables within goal setting. The first goal option here was to add more fruit or vegetables to a specific meal during the day, e.g., add berries to porridge at breakfast time
	Goal C2: Healthier food swaps	Swapping foods is commonly used in public health interventions, such as campaigns in the United Kingdom (Change4Life) and Australia,^50^ though we identified no literature around using this approach for individual goal setting. Adding fruit and veg was included as an ‘easy’ way to incorporate into the current diet
Cut down fast food regular take away/fast food was highlighted as problematic by the facilitators	Goal D1: Reduce weekly take aways by one	Cut down the number of take aways eaten during the week by one, rather than to abstain completely. Much of the research in this area refers to reducing advertising and access; as such, the first goal was agreed on by the research team and staff stakeholders
	Goal D2: Replace take away with home‐cooked version	The second goal to encourage home cooking was informed by existing service suggestions, which was supported by free, local cooking classes (recipes provided)
Drink more water, added following stakeholder involvement	Goal E1: Swap one caffeinated drink with a noncaffeinated alternative	Although the Eatwell guide^34^ states that tea and coffee contribute towards the recommended daily 6–8 glasses of water, evidence suggests that drinking more water can result in increased weight loss^51^, ^52^
	Goal E2: The second goal was to carry a water bottle	The second goal was to carry a water bottle, with ways to flavour water such as including fresh fruit. This was to address service users' complaints that they did not like the taste of water and based on existing recommendation by service
Choose my own		Facilitators felt that some service users would not like to choose predefined goals, and in response, we included a separate blank section to allow users of the intervention to define their own goals with two section: ‘what will I do?’ and ‘how will I do it?’

Including the BCT behavioural commitment was supported by our systematic review, which suggested that they showed promise in relation to changing dietary behaviours in the short term.^40^ Of the three randomised trials (409 participants) in the meta‐analysis, interventions that included a commitment device increased short‐term weight loss by a mean of 1.5 kg (95% confidence interval: 0.7, 2.4).

### 3.8 Mode of delivery

Discussions around development of an app occurred both within the research team and with stakeholders, but did not fulfil the APEASE criteria in terms of affordability (research development costs), practicability (number of those able to use apps, e.g., older population) and equity (smartphone owners). We decided that a paper booklet as an add‐on tool to the current course was most suitable, containing visual content and requiring limited writing, in response to the low literacy and language barriers identified in our qualitative work.

### 3.9 Checking stage

In addition to the water consumption goal added by stakeholders, a suggestion was made to revise the fat intake goals to fit with recommendations (5%), and to highlight only one goal was required. Following these changes, the intervention was finalised (Supporting Information Material 1) and summarised following the standard intervention reporting criteria TIDieR^53^ (Supporting Information Material 2).

## 4 DISCUSSION

This is the first paper to report the systematic development of an intervention targeting dietary behaviours within a low SES setting following the BCW. We have presented in detail the development of the SMART‐C intervention, a goal‐setting and behavioural contracting booklet targeting dietary behaviours for use within community‐run weight loss groups in low SES areas. To improve dietary behaviours within this context, we identified a need to target engagement with goal‐setting behaviours. Results from a COM‐B analysis identified that for this to happen, changes needed to occur within psychological capability, physical opportunity and reflective motivation for the service users, and social and physical opportunity for the staff.

The existing literature and our qualitative results identify the following barriers to adherence to lifestyle behaviours: understanding/knowledge, cognitive functioning (e.g., memory), low motivation, cost, time, access and social influences.^22^, ^54^, ^55^ In addition, we found barriers around commitment, as well as the importance of language and literacy considerations. One barrier not identified in our data was issues around mood states, which has been previously identified as a barrier to adherence, but better baseline mood is also a predictor of adherence.^22^, ^54^ This may require further exploration in future studies.

### 4.1 Strengths and limitations

This is the first paper that has used the BCW to design an intervention targeting dietary behaviour in low SES groups. Our intervention was responsive to the priorities of the local health economy, taking close account of context,^31^, ^56^ and is required, given that the ‘one size fits all’ approach clearly does not work. Though designed for a particular population/service, SMART‐C has the potential to be generalised across other public health settings for low SES populations, specifically in similar contexts for example, services that are free and locally available, not illness‐condition‐focused, with no healthcare professional involvement. Goal content was based on evidence, current guidelines and the needs of the population, and as such is population‐specific, rather than service‐specific. However, as identified previously in relation to tailoring interventions,^21^ the approach and content may not be appropriate for more culturally diverse groups, or for other groups such as those targeting male‐only attendees for whom alternative, effective approaches have been identified.^57^

As others have highlighted,^58^, ^59^, ^60^, ^61^ following the BCW is a lengthy process, resulting in delays between identifying a need and implementing the intervention. An added benefit of the stakeholder involvement^41^ is that it aided the process in relation to decision making and ‘real‐life’ application, and has the potential for speeding up implementation of the final intervention, given that they have been involved in the process. A particular benefit of involving staff, in addition to the direct changes to the interventions, was that they facilitated recruitment of members of the public, which supports previous recommendations that working in partnership with community groups can facilitate recruitment of seldom heard populations.^62^

Involving staff and service users as both participants and stakeholders was a strength of this intervention design study. In some cases, facilitators' perceptions of service user knowledge and behaviour differed somewhat to the views of the service users themselves, suggesting that they may be inaccurate and subject to bias. However, this will have been countered to some extent by the service users' involvement.

Few intervention design papers using the BCW have included or described stakeholder engagement as part of the process. Stakeholder involvement contributed heavily to understanding the context throughout the intervention design process, and while this is as a strength, a more inclusive approach could have been adopted, given that stakeholders were consulted with at various points throughout the study (e.g. feedback on the research protocol, on first draft of the intervention, occasional questions) rather than involved continuously throughout. Other approaches could have involved stakeholders further, such as a community‐based participatory approach. The approach adopted in this study shares some commonalities with this approach, such as building on existing ‘strengths and resources within the community’ (p. 274), and balancing research with action for mutual benefit.^63^ The researcher retained the ‘power’ throughout the research process; however, it was not an equal partnership, and it was not a long‐term partnership approach. This may not have been achievable within the time and funding limitations of a PhD, particularly given that the researcher was not working as part of a wider team, and the researcher endeavoured to involve the stakeholders and contributors where possible.

This intervention was focused on increasing the engagement of those already involved in weight loss services; as such, it does not address how to increase motivation to engage in services in the first instance. Furthermore, we focused this intervention around the in‐group behaviour of goal setting, but that does not address the external factors identified, such as the availability of low cost and fresh foods, which would need attention at an environmental and social planning level. This supports the need for a whole systems approach to address this health inequality,^20^ such as subsidising healthy food to increase its consumption in deprived areas.^64^ Future research will assess the acceptability of SMART‐C to all users. Intervention acceptability is important for implementation and is recommended in the design of complex interventions as part of the feasibility and piloting stage.^31^

## 5 CONCLUSIONS

Through following The BCW guide,^28^ alongside stakeholder involvement, qualitative data and existing literature and recommendations, we have systematically developed a novel, practical and theory‐informed intervention. The SMART‐C goal setting and contract booklet has been specifically designed to enhance community weight loss groups within low SES areas specifically targeting dietary behaviours (portion size, food labels, consumption of fruit and veg, fast food and water). Our next step is to generate a body of evidence about its acceptability and potential for implementation within routine care.

## CONFLICT OF INTERESTS

The authors declare that there are no conflict of interests.

## AUTHOR CONTRIBUTIONS

Nia Coupe devised the research, analysed the data and drafted the manuscript, all with supervision and input from Sarah Cotterill and Sarah Peters. All authors contributed to and approved the final manuscript.

## ETHICS STATEMENT

Ethical approval was obtained from the University Research Ethics Committee at the University of Manchester (UREC ref. no 15564). Informed consent was obtained from all individual participants included in the study.

## Supporting information

Supporting information.

Supporting information.

## Data Availability

Data are available from the corresponding author at reasonable request. The SMART‐C intervention is supplied as Supporting Information Materials.
