# Supplementary material for: Enhancing community weight loss groups in a low socioeconomic status area: Application of the COM‐B model and Behaviour Change Wheel
Source: Health Expect. 2021 Aug 4;25(5):2043–55. doi: 10.1111/hex.13325 (PMC9615060; doi:10.1111/hex.13325)
Supplement: Supplementary file 1 — Supporting information. [file HEX-25--s002.pdf]

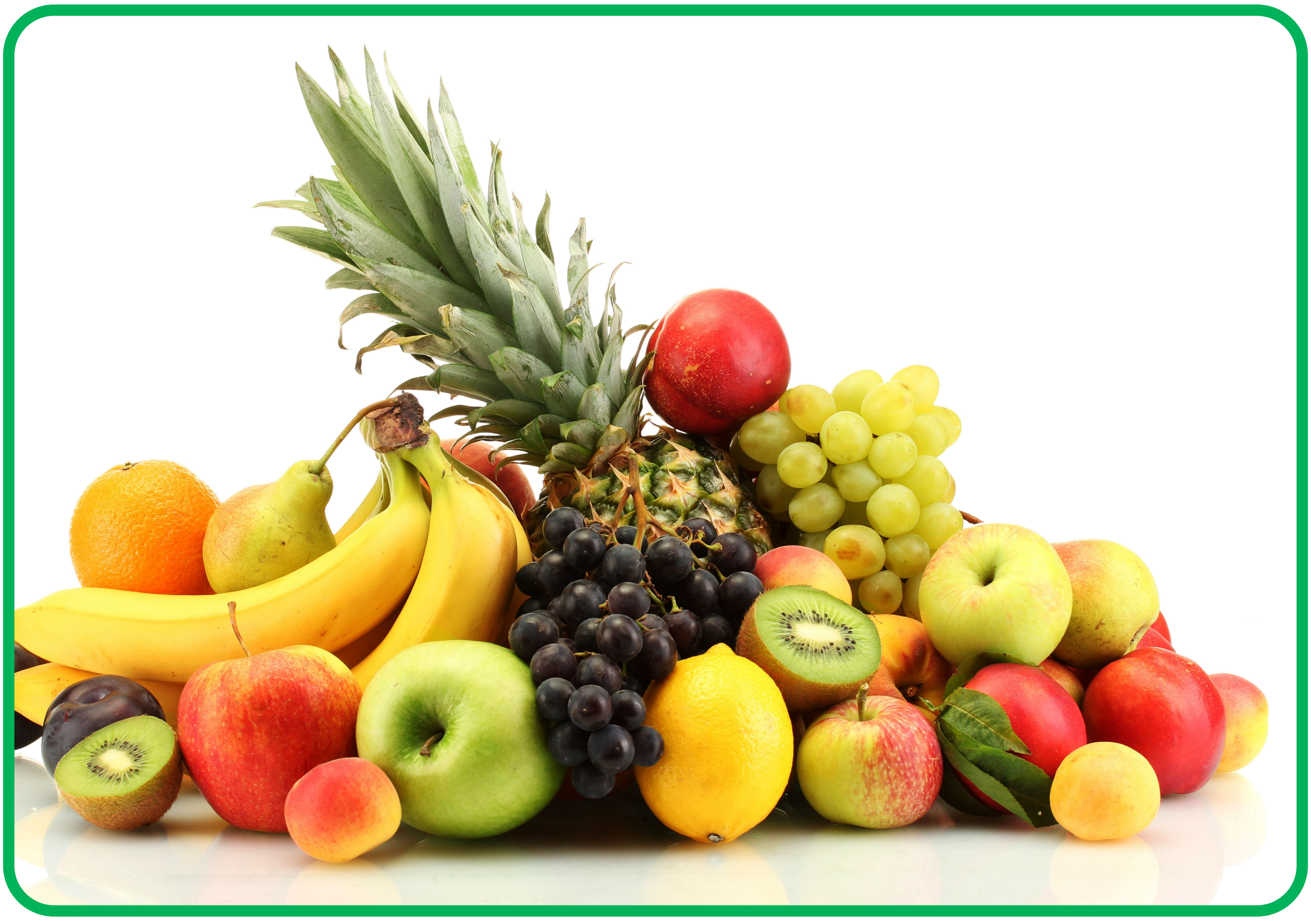

SMART Commitments

Small steps to healthier eating

[redacted] [redacted]

Contents

SMART Goals 1

Commitment Sheets 2

Portion Size 26

Food Labels 28

Fruit & Vegetables 30

Take-aways 32

Water 34

Recipes 36

How to use this booklet

This booklet has been developed to help you eat better *and* lose weight by setting SMART goals.

S	M	A	R	T
**Specific**	**Measurable**	**Achievable**	**Relevant**	**Timely**
Who, What, Where, When?	How much, how many?	Can you do it? Is it out of reach?	Is it worth-while?	Include a time limit

There are 4 main stages to goal setting in this booklet:

Commitment Sheet

1. Choose your goal (one of A-E, or make your own).
2. Decide on how many days you will do this.
3. **Commit** to the goal by signing your name.

Review

4. Revisit your goal from last week. If you've achieved your goal, try to make it more challenging, or try a new goal the following week.

Week 1 Commitment Sheet

Step 1 | ***This week I will...*** (choose ***ONE*** option from **A-F**)

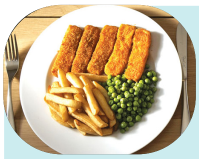

A

Eat smaller portions

(see page 26)

☐ *Use a smaller plate/bowl (circle) at breakfast/lunch/tea (circle) time*

OR

☐ *Use The Handy Guide at breakfast/ lunch/teatime (circle)*

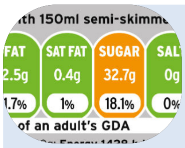

B

Check food labels

(see page 28)

☐ *Aim for more green than red labels when you shop.*

OR

☐ *Use the 5% fat rule*

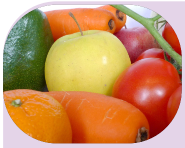

C

Eat more fruit & veg

(see page 30)

☐ *Add ________________ (fruit / veg) to my breakfast / lunch/ tea (circle)*

OR

☐ *Swap ____________ (unhealthy snack) for __________ (fruit/veg)*

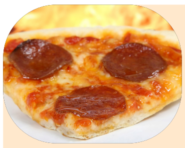

D

Cut down fast food

(see page 32)

☐ *Cut down from _______ to ________ take-aways / fast food meals*

OR

☐ *Cook my own*

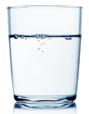

E

Drink more water

(see page 34)

☐ *Swap tea / coffee for fruit tea/ hot water or ___________________*

OR

☐ *Carry a water bottle with me*

F

Choose my own

(see page 1)

What will I do?

____

____

How will I do it?

____

____

Step 2 | *I will do this on...* (tick as many as you think you can do)

☐ Monday ☐ Tuesday ☐ Wednesday ☐ Thursday ☐ Friday

☐ Saturday ☐ Sunday

Step 3 | *Sign!*

Name: .... Date: ....

Witness: .... Date: ....

Week 1 Review

Step 4 | *Did you do it?*

- [ ] Yes, totally! → ***Great, well done!***
Why not make it more challenging this week, or add a new goal?

- [ ] Yes, a bit → ***Good effort!***
Why not try it again this week? What could you do differently?

- [ ] Not really → ***Not a problem.***
Why not make it a bit easier this week?

- [ ] No longer a goal → OK!
Why not try a different goal this week?

Notes

Now turn to Week 2 Commitment Sheet

Week 2 Commitment Sheet

Step 1 | ***This week I will...*** (choose ***ONE*** option from **A-F**)

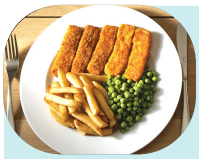

A

Eat smaller portions
(see page 26)

☐ *Use a smaller plate/bowl (circle) at breakfast/lunch/tea (circle) time*

OR

☐ *Use The Handy Guide at breakfast/lunch/teatime (circle)*

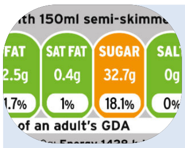

B

Check food labels
(see page 28)

☐ *Aim for more green than red labels when you shop.*

OR

☐ *Use the 5% fat rule*

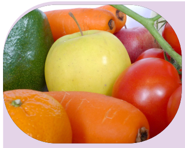

C

Eat more fruit & veg
(see page 30)

☐ *Add ________________ (fruit / veg) to my breakfast / lunch/ tea (circle)*

OR

☐ *Swap ____________ (unhealthy snack) for __________ (fruit/veg)*

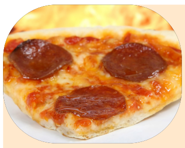

D

Cut down fast food
(see page 32)

☐ *Cut down from _______ to ________ take-aways / fast food meals*

OR

☐ *Cook my own*

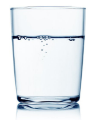

E

Drink more water
(see page 34)

☐ *Swap tea / coffee for fruit tea/ hot water or ____________________*

OR

☐ *Carry a water bottle with me*

?

F

Choose my own
(see page 1)

What will I do?

____

____

How will I do it?

____

____

Step 2 | ***I will do this on...*** (tick as many as you think you can do)

☐ Monday ☐ Tuesday ☐ Wednesday ☐ Thursday ☐ Friday

☐ Saturday ☐ Sunday

Step 3 | ***Sign!***

Name: .... Date: ....

Witness: .... Date: ....

Week 2 Review

Step 4 | *Did you do it?*

☐ Yes, totally! → ***Great, well done!***
Why not make it more challenging this week, or add a new goal?

☐ Yes, a bit → ***Good effort!***
Why not try it again this week? What could you do differently?

☐ Not really → ***Not a problem.***
Why not make it a bit easier this week?

☐ No longer a goal → OK!
Why not try a different goal this week?

Notes

Now turn to Week 3 Commitment Sheet

Week 3 Commitment Sheet

Step 1 **/** ***This week I will...*** (choose ***ONE*** option from **A-F**)

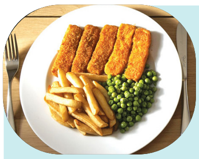

A

Eat smaller portions

(see page 26)

☐ *Use a smaller plate/bowl (circle) at breakfast/lunch/tea (circle) time*

OR

☐ *Use The Handy Guide at breakfast/ lunch/teatime (circle)*

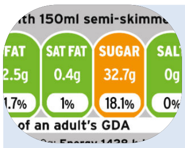

B

Check food labels

(see page 28)

☐ *Aim for more green than red labels when you shop.*

OR

☐ *Use the 5% fat rule*

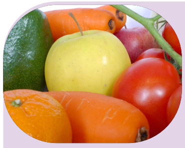

C

Eat more fruit & veg

(see page 30)

☐ *Add ________________ (fruit / veg) to my breakfast / lunch/ tea (circle)*

OR

☐ *Swap ____________ (unhealthy snack) for __________ (fruit/veg)*

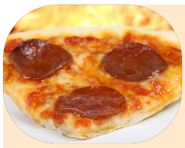

D

Cut down fast food

(see page 32)

☐ *Cut down from _______ to ________ take-aways / fast food meals*

OR

☐ *Cook my own*

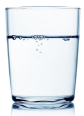

E

Drink more water

(see page 34)

☐ *Swap tea / coffee for fruit tea/ hot water or ____________________*

OR

☐ *Carry a water bottle with me*

? **F**

Choose my own

(see page 1)

What will I do? ______________________________

____

How will I do it? ______________________________

____

Step 2 | *I will do this on...* (tick as many as you think you can do)

☐ Monday ☐ Tuesday ☐ Wednesday ☐ Thursday ☐ Friday

☐ Saturday ☐ Sunday

Step 3 | *Sign!*

Name: .... Date: ....

Witness: .... Date: ....

Week 3 Review

Step 4 | *Did you do it?*

☐ Yes, totally! → ***Great, well done!***
Why not make it more challenging this week, or add a new goal?

☐ Yes, a bit → ***Good effort!***
Why not try it again this week? What could you do differently?

☐ Not really → ***Not a problem.***
Why not make it a bit easier this week?

☐ No longer a goal → OK!
Why not try a different goal this week?

Notes

Now turn to Week 4 Commitment Sheet

Week 4 Commitment Sheet

Step 1 / ***This week I will...*** (choose ***ONE*** option from **A-F**)

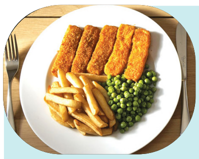

A

Eat smaller portions
(see page 26)

- ☐ *Use a smaller plate/bowl (circle) at breakfast/lunch/tea (circle) time*

OR

- ☐ *Use The Handy Guide at breakfast/ lunch/teatime (circle)*

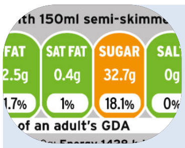

B

Check food labels
(see page 28)

- ☐ *Aim for more green than red labels when you shop.*

OR

- ☐ *Use the 5% fat rule*

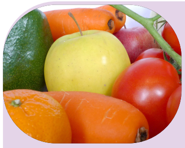

C

Eat more fruit & veg
(see page 30)

- ☐ *Add ________________ (fruit / veg) to my breakfast / lunch/ tea (circle)*

OR

- ☐ *Swap ____________ (unhealthy snack) for __________ (fruit/veg)*

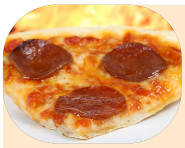

D

Cut down fast food
(see page 32)

- ☐ *Cut down from _______ to ________ take-aways / fast food meals*

OR

- ☐ *Cook my own*

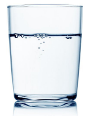

E

Drink more water
(see page 34)

☐ *Swap tea / coffee for fruit tea/ hot water or ___________________*

OR

☐ *Carry a water bottle with me*

? **F**

Choose my own
(see page 1)

What will I do?

____

____

How will I do it?

____

____

Step 2 | ***I will do this on...*** (tick as many as you think you can do)

☐ Monday ☐ Tuesday ☐ Wednesday ☐ Thursday ☐ Friday

☐ Saturday ☐ Sunday

Step 3 | ***Sign!***

Name: .... **Date:** ....

Witness: .... **Date:** ....

Week 4 Review

Step 4 | *Did you do it?*

☐ Yes, totally! → ***Great, well done!***
Why not make it more challenging this week, or add a new goal?

☐ Yes, a bit → ***Good effort!***
Why not try it again this week? What could you do differently?

☐ Not really → ***Not a problem.***
Why not make it a bit easier this week?

☐ No longer a goal → **OK!**
Why not try a different goal this week?

Notes

Now turn to Week 5 Commitment Sheet

Week 5 Commitment Sheet

Step 1 / ***This week I will...*** (choose ***ONE*** option from **A-F**)

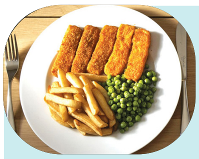

A

Eat smaller portions
(see page 26)

☐ *Use a smaller plate/bowl (circle) at breakfast/lunch/tea (circle) time*

OR

☐ *Use The Handy Guide at breakfast/ lunch/teatime (circle)*

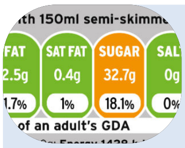

B

Check food labels
(see page 28)

☐ *Aim for more green than red labels when you shop.*

OR

☐ *Use the 5% fat rule*

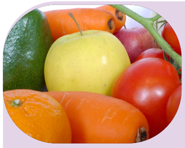

C

Eat more fruit & veg
(see page 30)

☐ *Add ________________ (fruit / veg) to my breakfast / lunch/ tea (circle)*

OR

☐ *Swap ____________ (unhealthy snack) for __________ (fruit/veg)*

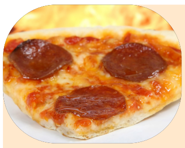

D

Cut down fast food
(see page 32)

☐ *Cut down from _______ to ________ take-aways / fast food meals*

OR

☐ *Cook my own*

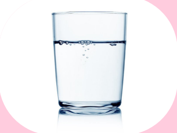

E

Drink more water
(see page 34)

☐ *Swap tea / coffee for fruit tea/ hot water or ___________________*

OR

☐ *Carry a water bottle with me*

?

F

Choose my own
(see page 1)

What will I do?

____

____

How will I do it?

____

____

Step 2 | ***I will do this on...*** (tick as many as you think you can do)

☐ Monday ☐ Tuesday ☐ Wednesday ☐ Thursday ☐ Friday

☐ Saturday ☐ Sunday

Step 3 | *Sign!*

Name: .... Date: ....

Witness: .... Date: ....

Week 5 Review

Step 4 | *Did you do it?*

☐ Yes, totally! → ***Great, well done!*** *Why not make it more challenging this week, or add a new goal?*

☐ Yes, a bit → ***Good effort!*** *Why not try it again this week? What could you do differently?*

☐ Not really → ***Not a problem.*** *Why not make it a bit easier this week?*

☐ No longer a goal → OK! *Why not try a different goal this week?*

Notes

Now turn to Week 6 Commitment Sheet

Week 6 Commitment Sheet

Step 1 / ***This week I will...*** (choose ***ONE*** option from **A-F**)

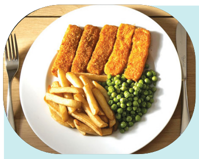

A

Eat smaller portions

(see page 26)

☐ *Use a smaller plate/bowl (circle) at breakfast/lunch/tea (circle) time*

OR

☐ *Use The Handy Guide at breakfast/ lunch/teatime (circle)*

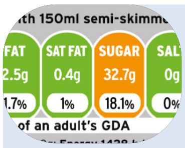

B

Check food labels

(see page 28)

☐ *Aim for more green than red labels when you shop.*

OR

☐ *Use the 5% fat rule*

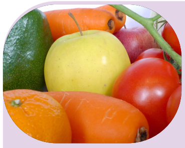

C

Eat more fruit & veg

(see page 30)

☐ *Add ________________ (fruit / veg) to my breakfast / lunch/ tea (circle)*

OR

☐ *Swap ____________ (unhealthy snack) for __________ (fruit/veg)*

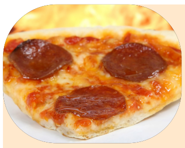

D

Cut down fast food

(see page 32)

☐ *Cut down from _______ to ________ take-aways / fast food meals*

OR

☐ *Cook my own*

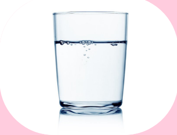

E

Drink more water

(see page 34)

☐ *Swap tea / coffee for fruit tea/ hot water or ___________________*

OR

☐ *Carry a water bottle with me*

?

F

Choose my own

(see page 1)

What will I do? ______________________________

____

How will I do it? ______________________________

____

Step 2 | ***I will do this on...*** (tick as many as you think you can do)

☐ Monday ☐ Tuesday ☐ Wednesday ☐ Thursday ☐ Friday

☐ Saturday ☐ Sunday

Step 3 | ***Sign!***

Name: .... **Date:** ....

Witness: .... **Date:** ....

Week 6 Review

Step 4 | *Did you do it?*

☐ Yes, totally! → ***Great, well done!***
Why not make it more challenging this week, or add a new goal?

☐ Yes, a bit → ***Good effort!***
Why not try it again this week? What could you do differently?

☐ Not really → ***Not a problem.***
Why not make it a bit easier this week?

☐ No longer a goal → OK!
Why not try a different goal this week?

Notes

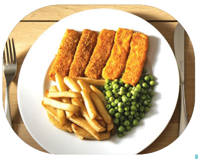

A. Portion Size

Even if you are eating the right kind of food, cutting down the portion size will help you lose weight. Here are two ways you can do this:

Option 1

Size Swap!

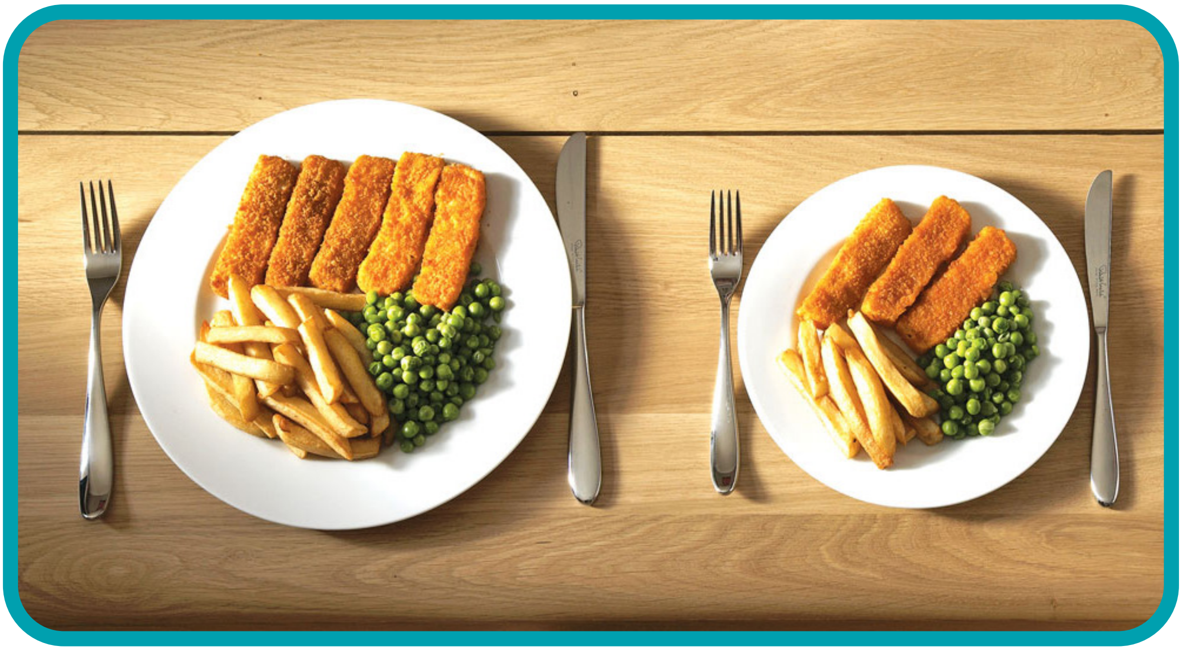

Use a smaller plate, bowl or glass to limit your portion. Either use ones you already own, or try buying smaller plates from a charity shop.

Option 2

The Handy Guide

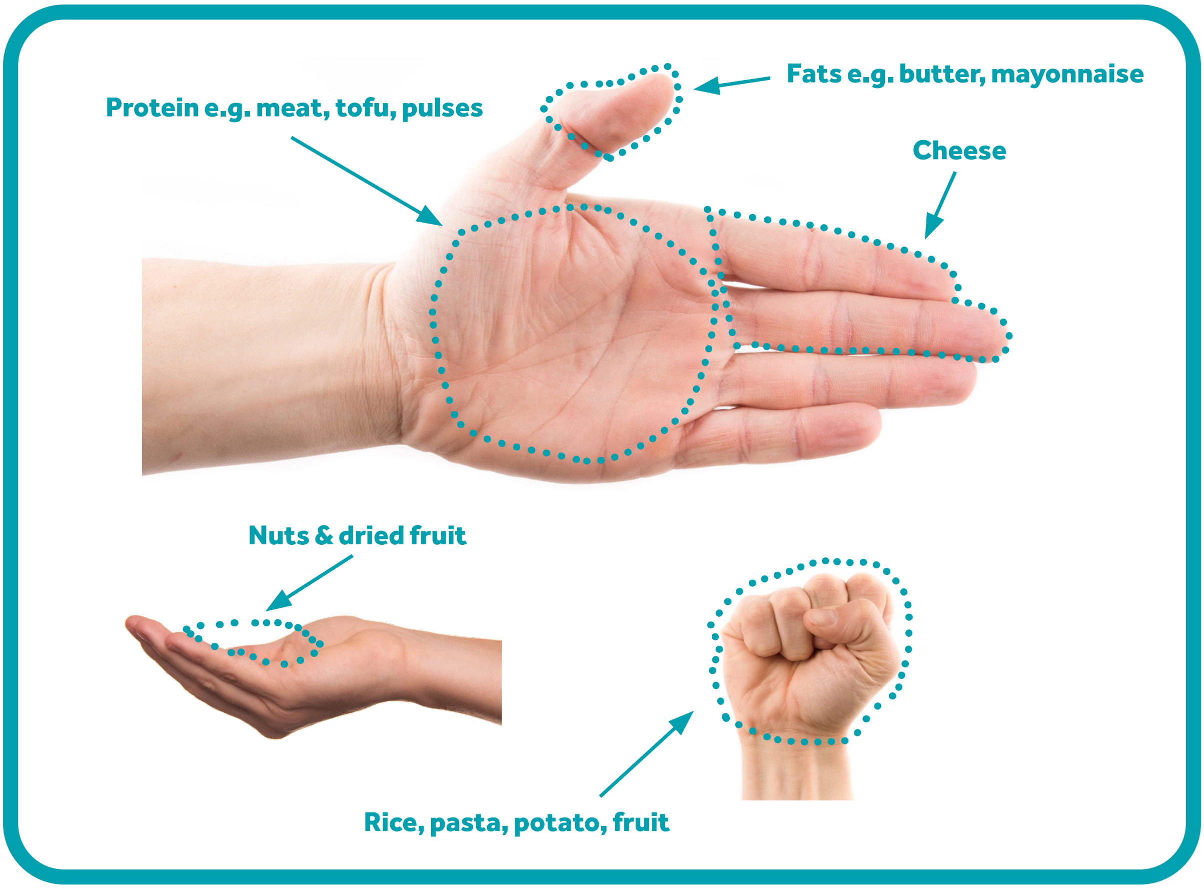

Use this quick hand guide to control your portions when serving or cooking your meals.

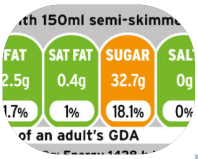

B. Food Labels

Looking at food labels when you shop can help you make healthier choices. Here are two ways you can use food labels:

Option 1
Colour Swap

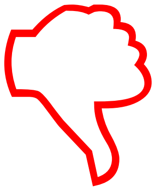

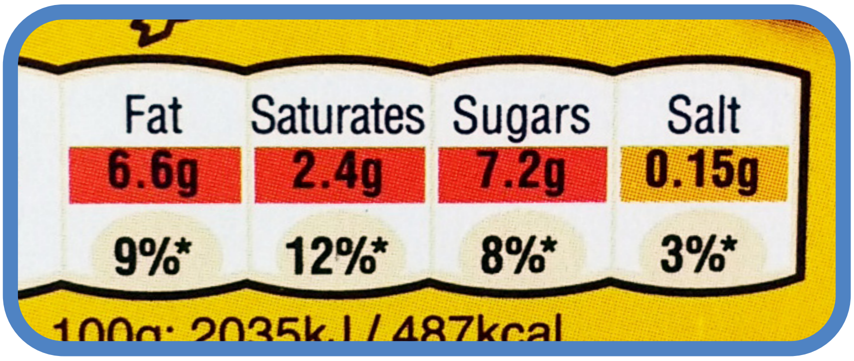

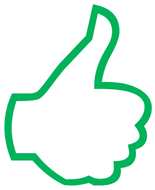

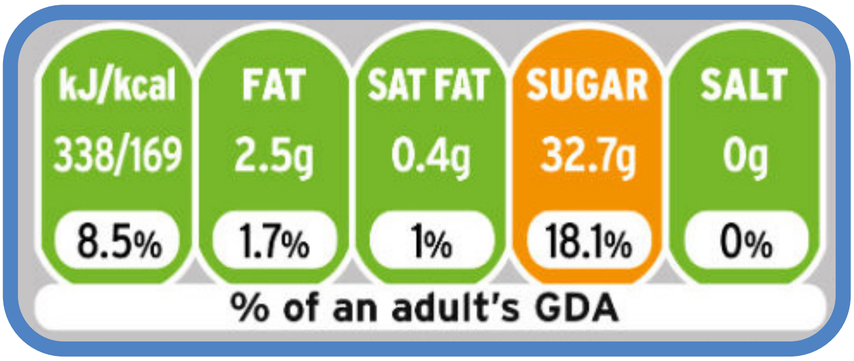

Cut down the number of foods you buy with RED labels and aim for more GREEN, and some ORANGE labels. Remember Green=Go, Red= Stop!

Option 2

Check Fat

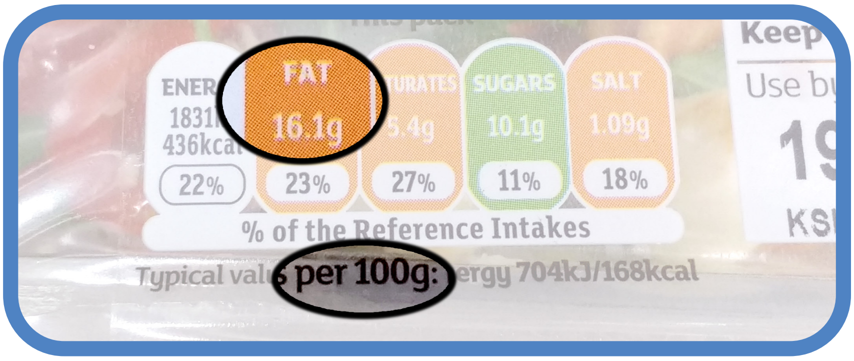

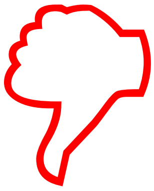

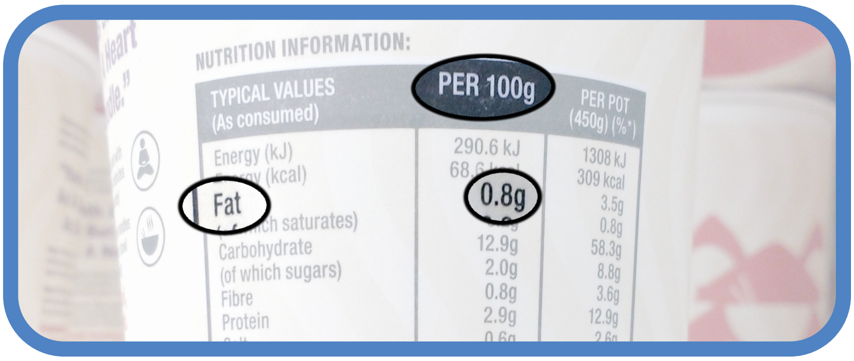

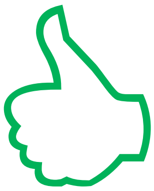

Use a rule of thumb when shopping- aim for less than 5% fat (5g per 100g). Keep your eye on the salt and sugar content too!

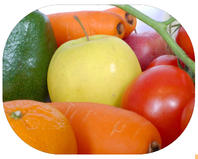

C. Fruit and Vegetables

Eating more fruit and veg can help you feel better and keep fuller for longer. Here are two ways to help you towards getting your 5-a-day:

Option 1
Add More

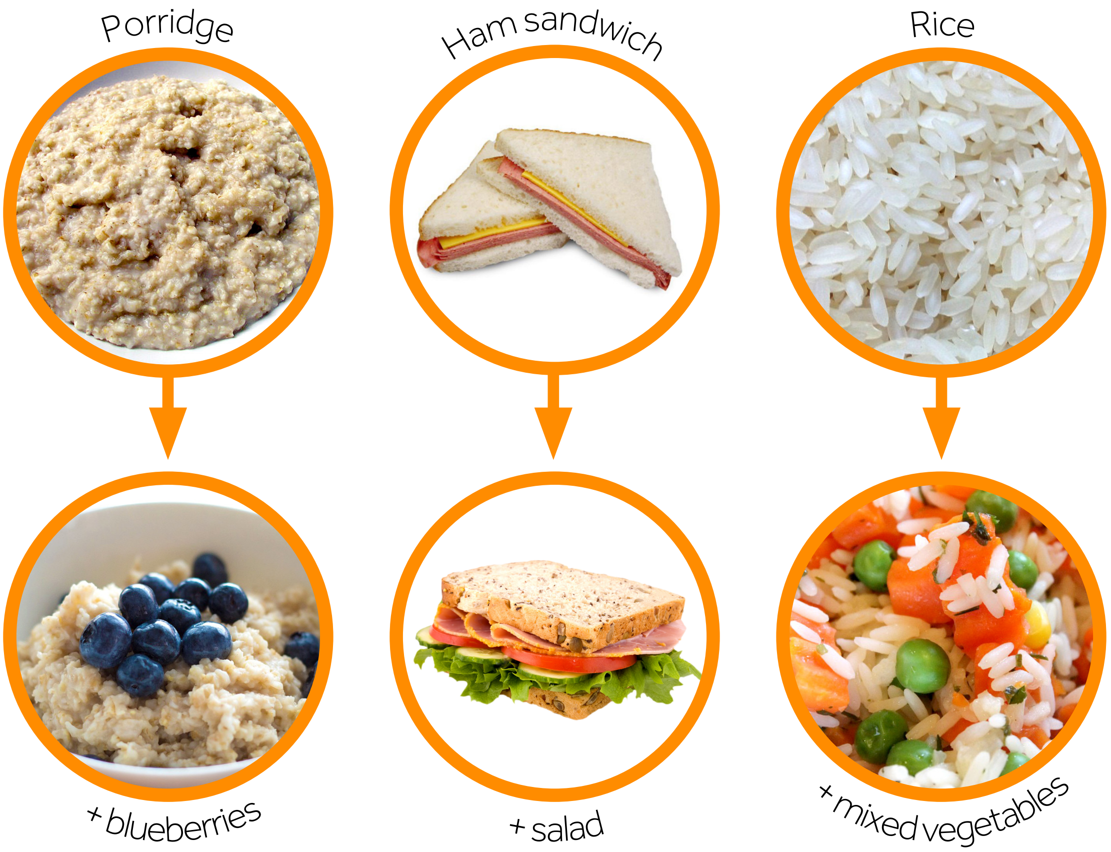

Add fruit or vegetables to your breakfast, lunch or dinner. Look at the suggestions above.

Top Tip

Dont forget frozen and tinned fruit and vegetables still count (as long as they're in their own juice or in water)

Option 2

Snack Swap

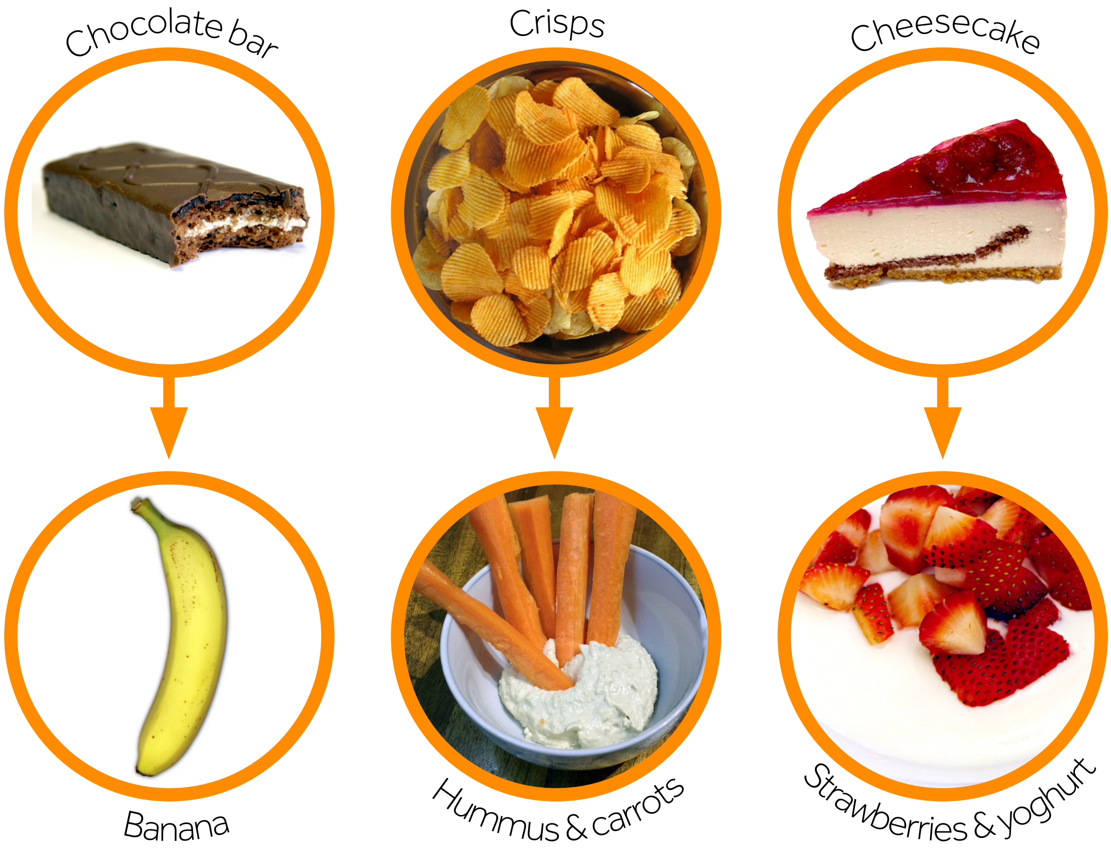

Swap that snack! Think of an unhealthy snack that you eat often, and swap it for a piece of fruit or a vegetable snack. Look at the suggestions above.

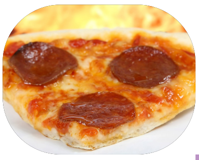

D. Fast Food

Take aways and fast food can be full of fat, sugar and salt which might be stopping you from losing weight. Here are two ways you can cut down:

Option 1
One Less

Week 1 – *Three take aways*

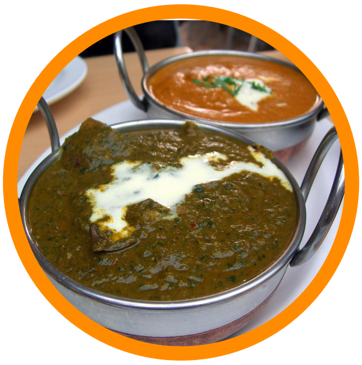
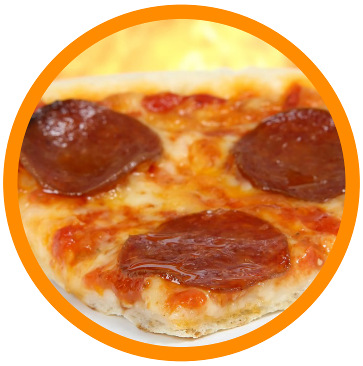
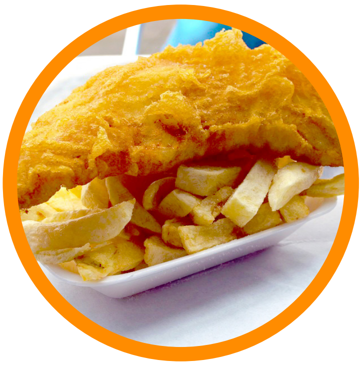

Week 2 – *Two take aways*

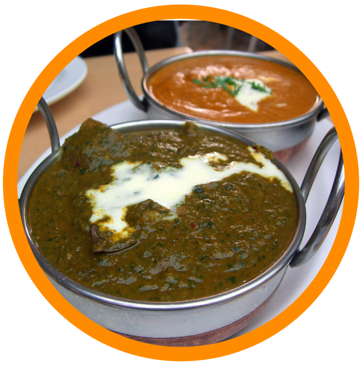
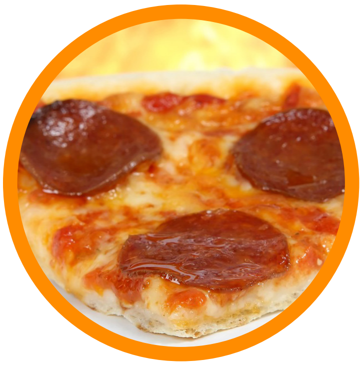

Count how many take away or fast food meals you had last week. Try cutting down that number this week by 1.

Option 2

Cook It!

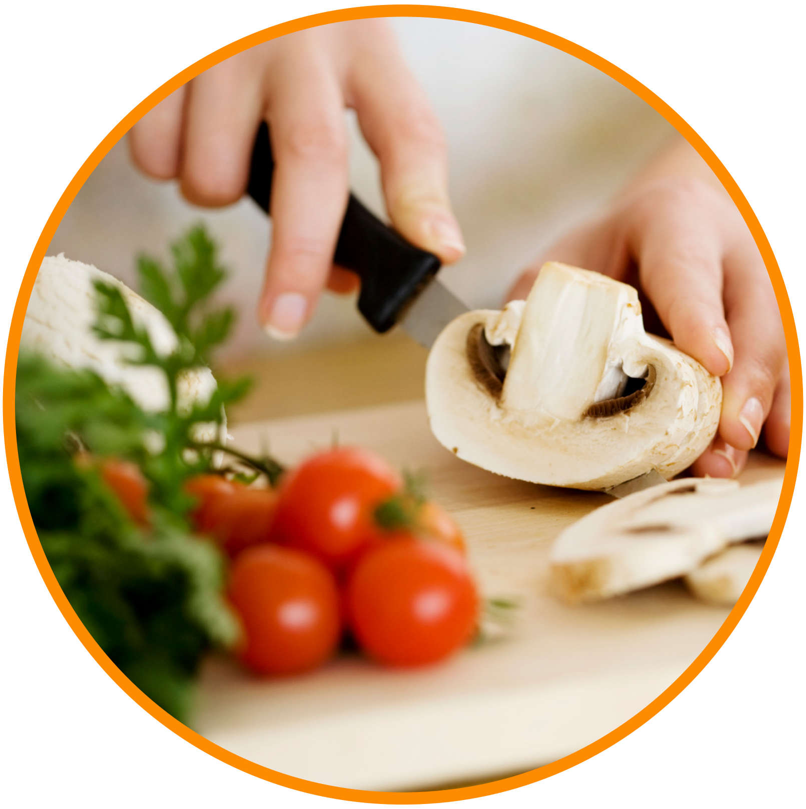

Miss the taste? Why not try cooking your own. There are plenty of easy healthy recipes you can try. Have a look online for some recipes and step by step videos (see back page), or turn to page 36 for some examples.

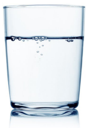

E. Water

We can consume a lot of calories through drink. Water has no calories and is important to keep us hydrated. Drinking more can make you feel better and help with your weight loss.

Option 1
Drink Swap

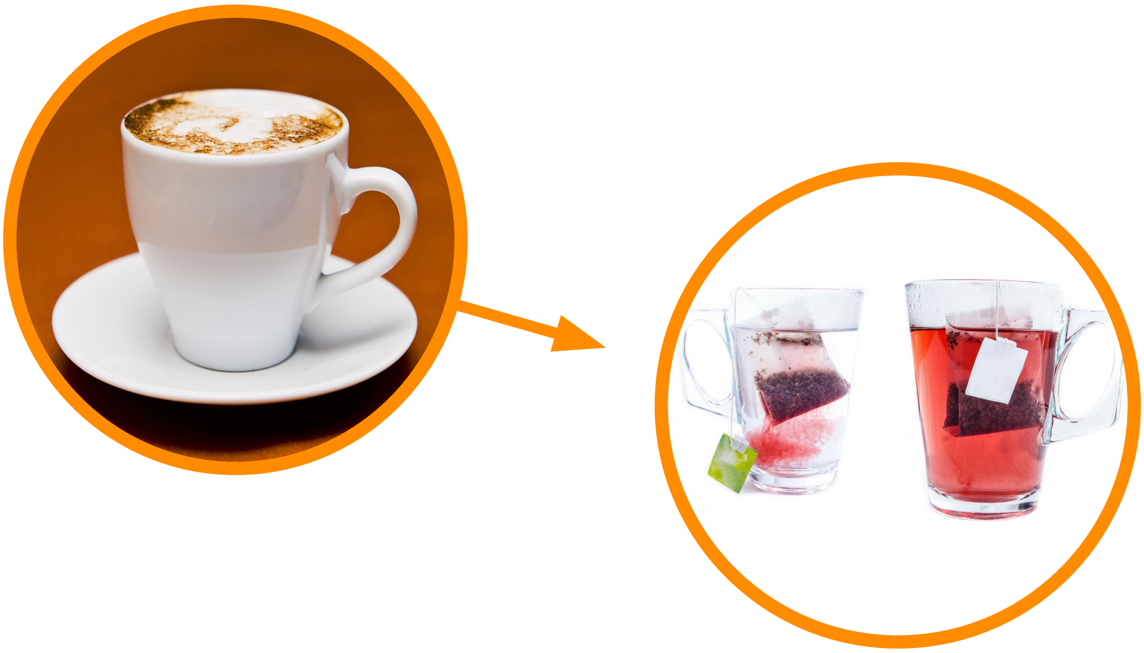

If you drink a lot of tea or coffee, try replacing one cup a day with a non-caffeinated alternative, such as fruit or herbal tea, or hot water with a slice of lemon.

Top Tip

Keep slices of frozen fruit in your freezer ready to flavour your water!

Option 2

Water Bottle

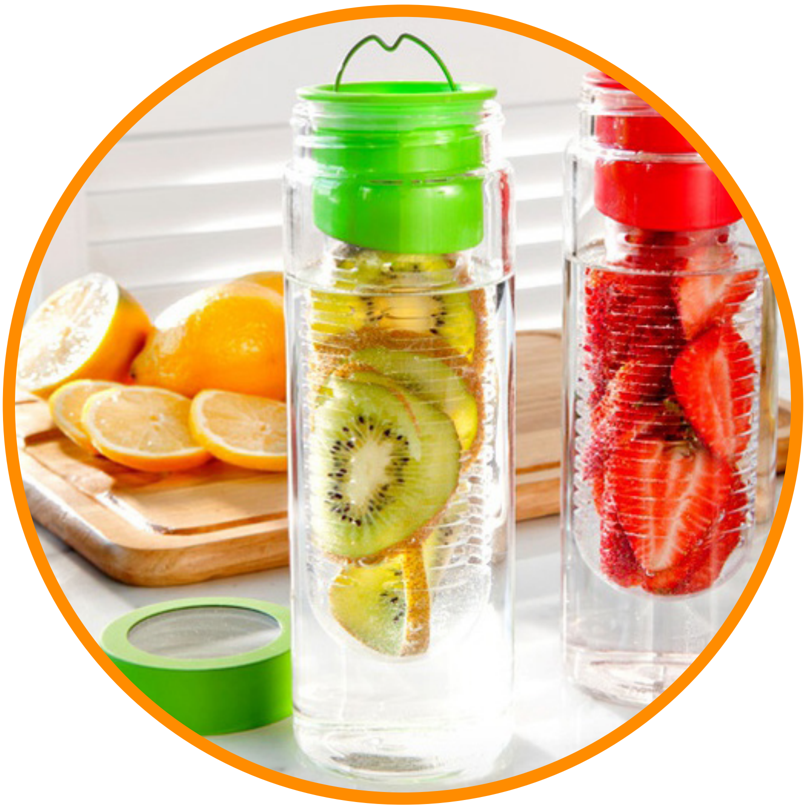

Take a bottle of water with you when you go out, or simply around the house. Don't like the taste of plain water? Try adding slices of fruit such as lemon, orange or lime.

Cook Your Own Take Away

Low Fat Chips

Ingredients

- Baking or sweet potatoes, peeled and cut into thick chips
- Low calorie spray e.g. Fry Light
- Salt and vinegar to serve

Method

1. Preheat your oven to 200C/180C fan/ Gas 6
2. Boil the chips for 4-5 minutes
3. Drain well and return to the pan and cover. Leave to cool slightly then shake the pan to roughen the edges.
4. Line a baking tray with baking paper and put the chips on top in a single layer. Spray with the low calorie spray and bake until golden, about 15-20 minutes.
5. Season with a little salt and vinegar, and enjoy!

Sweet and Sour Sauce

Ingredients

- 110g can pineapple in juice, chopped into chunks
- 5 tbsp vegetable stock
- 2 tbsp soy sauce
- 1 tbsp white wine vinegar
- 4 tbsp orange juice
- 1 tsp sweetener
- 11tspb tomato puree
- 2 tbsp finely diced red pepper
- 2 level tsp cornflour

Method

1. Place the pineapple chunks into the pan with the stock, soy sauce, vinegar, orange juice, sweetener, tomato puree and red pepper.
2. Bring to the boil and simmer gently.
3. Mix the cornflour with 2 teaspoons of water until smooth and stir into the sauce. Simmer for a few more minutes and serve with meat, veg and rice or noodles.

Chicken Korma

Ingredients

- 1 onion, roughly chopped
- 2 garlic cloves
- 2cm piece of ginger, peeled and roughly chopped
- 4 tbsp korma curry powder
- low calorie spray e.g Fry Light
- 4 chicken breasts, cut into bite sized pieces
- 500ml boiling chicken stock
- pinch of sweetener
- 150g fat free natural Greek yoghurt, whisked
- salt and freshly ground black pepper
- small handful of finely chopped fresh mint or coriander (optional)

Method

1. Blend the onion, garlic and ginger into a paste in a small food processor.
2. Fry the paste for 5 minutes in the cooking spray, then stir in the korma powder, cooking for 2 minutes.
3. Stir in the chicken and the stock and sweetener. Mix well, cover and simmer until the chicken is cooked through (10-12 minutes).
4. Remove from the heat, add yoghurt and seasoning.
5. Scatter over the chopped herbs if using, and serve hot with basmati rice.

Further resources

For more information about healthy eating and recipes, check out these websites:

- http://www.slimmingworld.co.uk/healthy-eating/recipes.aspx
- https://www.weightwatchers.com/us/recipe
- https://www.rosemaryconley.com/library/recipes
- https://www.bhf.org.uk/heart-matters/healthy-eating-toolkit/recipe-finder
- https://www.diabetes.org.uk/Preventing-Type-2-diabetes/im-at-risk-of-type-2-diabetes/

Or if you have a smart phone, try these free apps:

- Change4Life Food Scanner (Check how much sugar, fat, salt, and calories are in your food)
- Change4Life Smart Recipes
- One You Easy Meals

You can also ask your course leader about classes, courses and facilities in your area.

Credits

Photos:

www.freeimages.com / www.publicdomainpictures.net / pixabay.com /commons.wikimedia.org / fruitinfusion12.tumblr.com /aphunniblog.edublogs.com / clipartpanda.com / regalawnings.co.uk / healthyadvicer.com / psu.edu / shutterstock.com / imgur.com

Recipes:

http://www.slimmingworld.co.uk/healthy-eating/recipes.aspx

With thanks to all staff and members of the public who contributed to the development of this booklet
