# Supplementary material for: Enhancing community weight loss groups in a low socioeconomic status area: Application of the COM‐B model and Behaviour Change Wheel
Source: Health Expect. 2021 Aug 4;25(5):2043–55. doi: 10.1111/hex.13325 (PMC9615060; doi:10.1111/hex.13325)
Supplement: Supplementary file 2 — Supporting information. [file HEX-25--s001.pdf]

Electronic Supplemental Material 2. SMART-C TIDieR Checklist

**Brief Name** *(Intervention Components)*	**SMART-C booklet**		
	Stage 1. Goal setting	***Stage 2. Commitment making***	***Stage 3. Review goal***
**Why**<br><br>Rationale, theory or goal of the element	• Goal setting identified as 'active' component of weight loss intervention (Dombrowski et al, 2010).<br>• People struggle to set realistic goals, goals often not set in group ([redacted] al., 2018).	• Commitment making can improve outcomes, particularly to dietary goals ([redacted] 2019).<br>• Lack of commitment/ pressure identified in groups ([redacted] 2018).	• Goals often not revisited in groups, so don't know if achieved or if need adjusting ([redacted]., 2018)<br>• There is a want/need for ongoing monitoring ([redacted]., 2018).
**What**<br><br>Materials	• Booklet- (Commitment Sheet).<br>• Goals presented visually in the booklet using images as well as text, because of identified literacy/language barriers ([redacted] 2018).<br>• Given pre-set goals because of difficulty setting realistic goals, and an option to set their own as requested by F ([redacted] 2018).	• Booklet- Commitment Sheet.<br>• Commitment in form of a behavioural contract; space for signature of SU and witness (F) below goal setting section ([redacted]2019) in the booklet Commitment Sheets.	• Booklet- Review page<br>• Tick boxes provided to record if goal has been achieved or not.

**What**<br><br>Procedures	• F to provide booklet during 1st session and talk through goal setting options. Must highlight that only one goal should be chosen (PPI).<br>• Completed by SU, discussed with buddy (other SU within group), Supervised by F. Not recommended to choose spouse as buddy ([redacted] al., 2019)	• SU and F to sign commitment before end of session (goal setting and signature is therefore witnessed- [redacted] This also gives the F the opportunity to check suitability of goal.	• F to review goals during individual weigh in or during group discussions ([redacted] 2019)
**Who**<br><br>Expertise, background and training of provider.	• Facilitator must be familiar with health behaviour change and SMART goals.<br>• Training booklet provided for deliverers as requested.		
**How**<br><br>Mode of delivery	• Face-to-face delivery, in a group setting and in pairs to harness social support (Bukman et al, 2014)		
**Where**<br><br>Type of location	• Community venues such as community centres, gateway buildings, libraries.		
